# *Helicoverpa* genus on the edge of the continental U.S.: Flight phenology, analysis of hybrid presence, and insecticide performance in high-input field crops in Puerto Rico

**DOI:** 10.3389/finsc.2022.1010310

**Published:** 2022-11-03

**Authors:** Xiomara L. Flores-Rivera, Silvana V. Paula-Moraes, James W. Johnson, Cameron J. Jack, Omaththage P. Perera

**Affiliations:** ^1^ Syngenta Seeds, Salinas, Puerto Rico; ^2^ Entomology & Nematology Department, West Florida Research and Education Center, Jay, FL, United States; ^3^ Syngenta Crop Protection Inc., Greensboro, NC, United States; ^4^ Entomology & Nematology Department, University of Florida, Gainesville, FL, United States; ^5^ Southern Insect Management Research Unit, USDA Agricultural Research Service, Stoneville, MS, United States

**Keywords:** Sex pheromone trapping, hybrids of *Helicoverpa* sp., invasive lepidopteran pests, Puerto Rico, IPM, IRM

## Abstract

The genus *Helicoverpa* includes several agricultural pests globally. *Helicoverpa armigera* was reported in several countries in South America in 2013, and in Puerto Rico, in 2014. This territory is considered an agricultural hub, with a high-input system of seed production in the southern region of the island, and also at the edge of the continental U.S. Possible natural dispersion of populations of *H. armigera* from the Caribbean or other Central American regions poses a continuing risk to the U.S. This study was performed during the post-detection scenario of *H. armigera* in Puerto Rico, from 2018 to 2021. A year-round pheromone trapping program of adult males indicated an increase in the population from October to March and differences in the occurrence of *Helicoverpa* spp. between the municipalities Juan Diaz and Salinas. The proportion of *H. armigera*/*H. zea* and detection of congeneric hybrids between these species were assessed based on genital morphology and DNA analysis. Interestingly, neither *H. armigera* nor expected hybrids were detected in the present study. The susceptibility of *H. zea* populations to the insecticides Spinetoram, Emamectin benzoate, Chlorantraniliprole, and Esfenvalerate was assessed, and an overall significant effect of insecticide susceptibility was detected. Chlorantraniliprole and Emamectin benzoate had the highest efficacy. These results contribute to the Integrated Pest Management and Insect resistance management programs to *Helicoverpa* spp. in Puerto Rico. In addition, provide validated information to be considered in mitigation plans, in the scenario of an invasion of *H. armigera* in the continental U.S.

## Introduction

A review of the subfamily Heliothinae (Lepidoptera: Noctuidae), a cosmopolitan group of noctuid moths proposed the genus *Helicoverpa* (1), which differentiated the species *H. armigera* (Hübner) and *H. zea* (Boddie) (Lepidoptera: Noctuidae) based on male genitalia morphology. The larvae and adults of both species have similar morphological characteristics and precise identification could only be based on male genitalia dissection (1, 2), and more recently, molecular approaches (3–5).

Previously, these species were allopatric occurring in separate geographical regions, with *H. zea* considered to be derived from *H. armigera* due to a genetic bottleneck that occurred 2 million years ago (4, 6). *Helicoverpa zea* is found throughout the Americas (1). In the United States, more than 30 crops are the host for this species and is considered to be one of the most important pests in row crops, southern U.S., including cotton (7). *Helicoverpa armigera* is reported to have a broader host range than *H. zea* (1, 8) and is widespread in Africa, Europe, Asia, and Oceania (9). This species is considered a major pest of food, fiber, and oil crops and has been reported in more than 67 host plant families, including Asteraceae, Fabaceae, Malvaceae, Poaceae, and Solanaceae (8).

Data from 1,208 interceptions of *H. armigera* in the international trade of commodities from 77 countries, including the Netherlands, Israel, and North Africa reported the association of this pest with food plants and cut flowers (10). *Helicoverpa armigera* was first reported in Brazil causing outbreaks during the 2012-2013 crop season, in commercial fields of cotton, corn, soybean, tomato, and beans, among other host plants (11, 12). A follow-up study recovering specimens from previous collections in the country and performing genitalia dissections (2), and PCR-RFLP molecular analysis following Behere et al. (3) indicated that this species was already present in the south region of Brazil at least before October 2008 (13). Further detections of this species were reported in Paraguay (14), Argentina (15), Uruguay (16), Colombia, Peru, Surinam, and the Dominican Republic (17).

The potential of invasion of this species in the continental U.S. was modeled and indicated the risk of natural dispersal from Caribbean islands or Mexico, due to the existence of suitable climate and extensive areas of host crop plants, especially in the southern U.S., with an estimated impact of US$ 78 billion p.a (18). In Puerto Rico, *H. armigera* was reported by the Plant Protection and Quarantine program (PPQ) (19) as a result of the USDA survey effort in 2014 (20). Phylogeographic analyses were performed based on comparing several haplotypes of 171 specimens of *H. armigera*, from 27 countries (10). These analyses included the three specimens intercepted in 2015 during survey trapping in south Florida (21), one year after detection in Puerto Rico. The results of these analyses could not determine the origin of the *H. armigera* population detected in both Puerto Rico and the three specimens in Florida (10). A study conducted between February 2016 and January 2017 in Puerto Rico identified four specimens of *H. armigera* in the sex pheromone trapping (22). However, it was detected at a low occurrence of *H. armigera* and a high abundance of *H. zea*, which can suggest that the invasion of this species was in its early stage (23).

Puerto Rico is considered an agricultural hub, with a high-input system of seed production in the southern region of the island, due to the tropical climate that allows extended crop season (24). This region is also on the edge of the area of the continental U.S., which possible natural dispersion of populations of *H. armigera* from the Caribbean or other Central American regions to the continental U.S. poses a continuing risk. However, few studies have been performed on the populations of the genus *Helicoverpa* in the post-detection scenario of *H. armigera* in Puerto Rico (10, 22, 23). More information is needed to support management recommendations for the genus *Helicoverpa*, including the expected occurrence of hybrids between *H. zea* and *H. armigera*. The objectives of this study were: 1) Document the occurrence and seasonal flight of *H. armigera* in the host plants corn, soybean, and sunflower in the southern region; 2) Determine the possible occurrence of hybrids between *H. armigera* and *H. zea* caught in the pheromone trapping; and 3) Evaluate the performance of insecticides commonly adopted to manage this genus in high-input systems of seed production. The results of this work represent a contribution to the improvement of the IPM and Insect Resistance management of the *Helicoverpa* genus in Puerto Rico, considering the expected coexistence of the native *H. zea* with *H. armigera*. This study provides also data on the magnitude of occurrence and seasonal phenology of flight on the island, and the performance of insecticides currently adopted for the management of *Helicoverpa* spp. The results of this work also contribute with validated information for mitigation plans, in a scenario of *H. armigera* invasion in the continental U.S.

## Materials and methods

### Year-round trapping of *Helicoverpa* sp. in commercial fields

A continuous trapping program for *H*. *armigera* and *H. zea* was conducted from 2018 to 2021 in the municipalities of Salinas (17° 58’38.89” N, -66° 17’52.62” W) and Juana Díaz (18°03’8.86N, -66°30’23.62W) in Puerto Rico. These two locations were selected for the year-round pheromone trapping because this southern region of Puerto Rico concentrates a large production of vegetables and is one of the largest winter nursery breeding operations in the United States of soybean, sunflower, corn, cotton, and sorghum (24). A total of ten bucket traps were placed in commercial fields of corn, soybean, and sunflower, and 19 traps in open field areas during the fallow season, using green bucket traps (International Pheromone Systems, IPS, Vestaburg, MI), with the *H. armigera* sex ABW pheromone lure (Trece, Inc., Adair, OK). The field sizes vary from 0.5 to 2 acres and the criterion of one trap in average per acre was followed, in a total of five bucket traps placed in corn, two bucket traps in soybean, and three bucket traps in sunflower fields. In addition, six Texas cone traps (25, 26), and 13 Scentry Heliothis traps (Scentry Biologicals, Inc., Billings, MT) were set in the fields with the *H. zea* L215 sex pheromone lure (Scentry Biologicals, Inc., Billings, MT). Traps were positioned at least 100 m apart. Bucket traps were mounted around a 1.2 m above-ground wood stake, on the east edge side of each field (wind direction is southwest) to promote dispersion of pheromone scent into the fields. Pheromone lures were replaced every 3 weeks. Moth samples captured in the traps were collected weekly, stored in 26.8cm x 27.3cm Ziploc^®^ plastic bags (S. C. Johnson, Racine, WI), transported to the Syngenta Seed Production System Laboratory in Salinas, PR, and kept in an upright freezer (-18°C) pending subsequent genitalia dissection and molecular analysis for species identification.

### *Helicoverpa* spp. identification

An initial sample screening was performed for each pheromone trap collection to separate specimens of *Helicoverpa* spp. from other cross-attracted species. Moths of the genus *Helicoverpa* were identified based on the presence of the wing morphological characteristics, such as a black color spot on the forewing, the presence of a broad dark transverse band distally, and hind wings lighter in color (1). A subsample of 76 specimens of *Helicoverpa* spp. collected from corn fields in Juana Díaz were then dissected for identification based on male genitalia morphology (2). The criteria for selection of the subsamples were based on morphological characteristics and the moths were collected from traps placed near corn fields in the reproductive stage (R1-R2). Each specimen was identified by a code, and the abdomen of the subsample of the *Helicoverpa* moths was removed using forceps and placed in 70% isopropyl alcohol for approximately 2 minutes to re-hydrate, before transferring to an individual 20mL-glass vial filled with 10 mL of a 10% potassium hydroxide solution (10% KOH). The abdomens were heated to 50°C for 45 minutes. After 45 minutes the KOH was removed using a dropper, and the abdomens were rinsed with alcohol (Brambila 2009). The abdomen was placed in a petri dish using a 7X-45X Simul-Focal Trinocular Zoom Stereo Microscope (AMScope, Irvine, CA, USA) and the male genitalia was extruded, using a fine point, straight tip, stainless forceps (BioQuip, Rancho Dominquez, CA, USA), applying light pressure from the base to the apex of the abdomen to extrude the genitalia. A fine paintbrush (Walmart^®^, Santa Isabel, PR) was used to brush clean the aedeagus before looking at the diagnostic characteristics. The diagnostic genitalia characteristic used for the identification of *Helicoverpa* spp. followed Pogue (2), considering the number of small lobes at the base of the vesica, near the apex of the aedeagus. A specimen with three lobes was identified as *H. zea*, and a specimen with a single lobe was identified as *H. armigera*, following Pogue (2).

To determine the genetics of the *Helicoverpa* spp. samples were collected, a subsample of 550 specimens was selected and DNA analysis was performed in the Entomology Laboratory at West Florida Research and Education Center, Jay, Florida. DNA was extracted from individual moths following the manufacturer’s instructions, using Qiagen Blood and tissue kit (cat. #65506). The PCR-based method was used for species identification of *H. zea* and *H. armigera* using the three-primer cocktail high-resolution melt curve (HRM) method developed by Perera et al. (5). DNA samples were subjected to PCR amplification with the three-primer cocktail and the amplicons were resolved on a 1% agarose gel. Six specimens with two amplicons were tentatively designated as hybrids and were submitted to sequence analysis in the Southern Insect Management Research Unit, USDA, ARS, Stoneville, MS. Ribosomal RNA gene region of approximately 1300 bp containing the internal transcribed spacer (ITS) 1, 5.8S rRNA subunit, and ITS2 was amplified from DNA extracted from the putative hybrid insects and control *H. zea* using a forward primer designed to 18S (310; 5’- ATCATTTAGAGGAAGTAAAAGTCGTAACAAGGT -3’) and a reverse primer designed to 28S (387; 5’- TTCCTGTTCGCTCGCCGCTACT-3’). Amplicons were resolved on a 0.8% agarose gen and the DNA bands excised from the gel were purified using QiaX gel purification reagents following the manufacturer’s instructions (Qiagen). The resulting DNA fragments were cloned into PCR2.1 vector using TOPO TA cloning kit (Invitrogen) and 12 recombinant colonies representing each insect were submitted to USDA ARS Genomics and Bioinformatics Research Unit, Stoneville, MS for Sanger dideoxy sequencing. ITS 1 and ITS2 nucleotide sequences from the suspicious hybrids from the PCR-based method and control insects were aligned with the respective sequences from *H. zea* and *H. armigera* to determine the source species.

### Insecticide susceptibility bioassays

The insecticide susceptibility of populations of the *Helicoverpa* genus to pyrethroid, avermectin, spinosyn, and diamide were documented in populations collected in corn ears, in Salinas, and in Juana Diaz. Around 150 larvae per location, ranging from 2^nd^ to 4^th^ instars were collected to establish field-derived colonies to be used in bioassays. The bioassays were conducted in the Syngenta Seed Production System Laboratory in Salinas, PR. Larvae were collected from corn fields in each municipality (trap crop and field corn) during the 2021 crop season. Larvae of *Helicoverpa* spp. were identified based on the presence of spines on the body of the larvae to distinguish this genus from *Spodoptera frugiperda*, another prevalent species associated with corn in the region. In addition, a subsample of 10 insects from the field-derived colonies from each municipality was submitted to DNA analysis as previously described for species identification and validation of the species identity of the colonies. The field-derived colony was established by placing each larva collected in field in a 71 grams souffle plastic cup containing an all-purpose Lepidoptera diet (Frontier Agricultural Sciences, Newark, DE) and transported to the seed production system laboratory in Salinas. Larvae were maintained at 25 ± 1°C, 40% relative humidity, and 12 h:12 h, light: dark photoperiod. The pupae were placed in Petri dishes inside 3.8-liter plastic containers (ePackageSupply, Evansville, IN) and used as mating cages. White cotton cloth was placed at the top of the container to serve as an oviposition surface. The moths were fed with a 20% sucrose solution change every two days. The egg sheets were collected daily and placed in a 9.4-liter rectangular plastic container (Rubbermaid food storage container). Approximately 200 neonates were transferred to 71 grams souffle plastic cups with an all-purpose Lepidoptera diet from each population and reared until they reached 3rd instar and had the appropriate size for the performance of the bioassays.

The bioassays with esfenvalerate, emamectin benzoate, and spinetoram were performed following Da Silva et al. (23), in which bioassay cups on 30-well trays were filled with 1 mL of artificial moth diet per well and insecticides dilutions applied on the diet surface. After 30 minutes, one single larva was placed inside the well

Diet overlay bioassays using 128-well trays (Frontier Agricultural Sciences, Newark, DE) were filled with 1 mL of general-purpose lepidopteran diet (Frontier Agricultural Sciences, Newark, DE). Once the diet was solidified and cool, 20µL of the insecticide concentration with 5% of a surfactant was dispensed on top of the diet, covering the entire surface of the 1.5 cm^2^ well. After the solution had dried, a single third instar *Helicoverpa* spp. larva was placed on top of the diet using a fine touch painting brush (Walmart^®^, Santa Isabel, PR).

Insecticide dilutions of insecticides registered to manage *Helicoverpa* sp. in the region were selected for this study. The high label rate per acre of commercial formulations of the insecticides Esfenvalerate (Asana^®^ XL, Valent^®^), Emamectin benzoate (Proclaim^®^, Syngenta^®^), Spinetoram (Radiant^®^ SC, Corteva Agriscience™) (27), and Chlorantraniliprole (Coragen^®^, FMC Ag US), were prepared in distilled water with adjuvants (**Table 1**). Four repetitions of 12 larvae for each product rate were prepared, plus a control group that consisted of a general-purpose diet and a solution of distilled water with a 5% surfactant. Larval mortality was assessed at 48 h and the number of dead larvae was recorded. The bioassay with chlorantraniliprole followed the Insecticide Resistance Action Committee (28) Method Number 20, as is recommended by the IRAC Diamide Working Group for evaluating the susceptibility status of diamides insecticides. The 128-well bioassay trays (Frontier Agricultural Sciences) were filled with 1g of stonefly Heliothis premix diet (Educational Science, League City, TX) mixed with the insecticide dilution and surfactant. The number of replications and quantity of larvae for the diet incorporated bioassay were the same as for the diet overlay bioassay previously described. Daily inspections indicated that 100% of larvae were dead after 48 h of exposure, but final larval mortality was assessed on day 7.

**Table 1:** Commercial insecticides and label rate tested in susceptibility bioassays to document *Helicoverpa* sp. populations from Salinas and Juana Diaz, Puerto Rico. 2021 crop season.

Chemicalgroup	ActiveIngredient	Commercialname	IRACGroup	Label ratefor field crop
Pyrethroid	Esfenvalerate	Asana XL^®^	3A	5.8-9.6fl oz/ac
Spinosyn	Spinetoram	Radiant SC^®^	5	3-6fl oz/ac
Avermectin	Emamectin Benzoate	Proclaim^®^	6	2.4-4.8fl oz/ac
Diamide	Chlorantraniliprole	Coragen^®^	28	3.5-7.5fl oz/ac

### Statistical analysis

The residual plots in SAS were used to test the best fit of the data distribution of the pheromone trapping data, and the Poisson distribution provided a better fit. The effect of the municipality and month in the total number of *Helicoverpa* spp. moth caught were analyzed using a Generalized linear mixed model (GLMM) on SAS (version 9.4). Due to the nested nature of the pheromone trapping, crop and location were included in the model as nested random effects. A significant interaction was detected between the trapping month and the municipality, the means were compared using the Tukey’s HSD test (p-value= 0.05). The percentage mortality for each insecticide was calculated and corrected considering the mortality of control in each bioassay, following Abbotts’ formula. Differences in the insecticide susceptibility of *Helicoverpa* sp. populations were tested using LS Means’ S test (p-value = 0.05).

## Results

### Year-round trapping of *Helicoverpa* sp. in commercial fields

**Figure 1:**
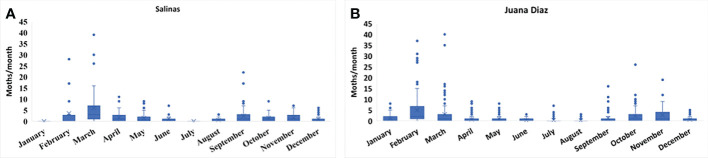
Total number of moths of *Helicoverpa* spp. in sex pheromone traps using *H. armigera* lure from 2018 to 2021. **A:** Collection in the municipality of Salinas, Puerto Rico. **B:** Collection in the municipality of Juana Diaz, Puerto Rico.

**Figure 2:**
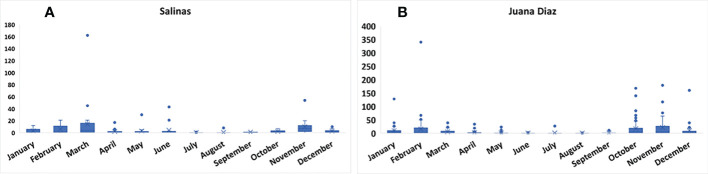
Total number of moths of *Helicoverpa* spp. in sex pheromone traps using *H. zea* lure from 2018 to 2021. **A:** Collection in the municipality of Salinas, Puerto Rico. **B:** Collection in the municipality of Juana Diaz, Puerto Rico.

The continuous pheromone trapping of *Helicoverpa* spp. in high-input systems cultivated with corn, soybean, and sunflower in the municipalities of Salinas (**Figure 1A**) and Juana Díaz (**Figure 1B**) in Puerto Rico indicated flight throughout the year, with a high occurrence of *Helicoverpa* spp. moths from October to March. Between the 2018 to 2021 crop seasons, a total of 1,835 moths were caught in the trapping with *H. armigera* pheromone lure in Juana Díaz, and 734 moths were caught in the municipality of Salinas. The trapping performed with *H. zea* pheromone lure indicated the same pattern of a flight of *Helicoverpa* spp. in the regions under study (**Figures 2A, B**). A total of 5,998 and 1,123 *H. zea* moths were caught in Juana Díaz and in Salinas, respectively. The effect of the municipality (Salinas and Juana Diaz), crop, and month on the abundance of *Helicoverpa* spp. were tested, and a significant interaction between municipality and month was detected (p-value <0.0001; F-Value=92.23). During the months from September to February, a high abundance of *Helicoverpa* spp. was detected in Juana Diaz (**Table 2**). Conversely, a high abundance of *Helicoverpa* spp. was detected in Salinas, during March, April, and May (**Table 2**). Morphological dissections of the male genitalia of a subsample from the trapping with *H. armigera* lure indicated that all the specimens had the presence of the three lobes at the base of the vesica and were identified as *H. zea*. The samples were analyzed using the HRM method validated by Perera et al. (5) initially indicated the possible existence of six hybrids of *H. armigera* x *H. zea* collected from corn fields and fallow area in Juana Diaz, and all remaining analyzed specimens were identified as *H. zea*. Subsequent nucleotide sequence analysis of ITS1 and ITS2 regions did not confirm the presence of *H. armigera* x *H. zea* hybrids in the collections, indicating that hybrids were not detected in any collection site.

**Table 2:** Simple effect comparisons of municipality and month on the abundance of *Helicoverpa* spp. caught in sex pheromone trapping using *H. armigera* lure in Salinas and Juana Diaz municipalities, Puerto Rico, from 2018 to 2021.

Month	Mean monthly abundance of *Helicoverpa* sp. (± SD)in municipalities of Puerto Rico^1^	
	Juana Diaz	Salinas
January	1.19 ± 1.86 a	0.00 ± 0.00 b
February	4.95 ± 7.19 a	3.55 ± 7.25 b
March	3.05 ± 5.80 b	6.00 ± 8.80 a
April	0.74 ± 1.55 b	1.67 ± 2.24 a
May	0.97 ± 1.57 b	1.43 ± 2.24 a
June	0.37 ± 0.66 b	1.00 ± 1.53 a
July	0.38 ± 0.89 a	0.00 ± 0.00 b
August	0.19 ± 0.51 b	0.55 ± 0.93 a
September	1.21 ± 2.63 a	2.44 ± 4.06 b
October	2.20 ± 3.40 a	1.49 ± 1.76 b
November	2.65 ± 3.59 a	1.57 ± 1.85 b
December	0.67 ± 1.20 a	0.89 ± 1.67 b

^1^The estimate means of moth abundance ± SE with the same letter, in the same row are not significantly different, p-value ≤ 0.05.

### *Helicoverpa* spp. identification

The PCR analysis of a subsample of insects from the field-derived colonies established in the laboratory from each municipality indicated the presence of only *H. zea*, which is consistent with the results from the sex pheromone trapping without the presence of hybrids.

### Insecticide susceptibility bioassays

The results of bioassays are interpreted as the susceptibility of *Helicoverpa* spp. populations. The populations from Juana Díaz and Salinas did not differ in insecticide susceptibility (**Table 3**). However, an overall significant effect of insecticide susceptibility was detected (p-value<0.001, F=87.20) (**Table 3**). Chlorantraniliprole and emamectin benzoate had statistically similar performance, with mortality higher than 90% (**Table 4**), followed by spinetoram with mortality above 70%, and esfenvalerate, which had the lowest performance when compared with the other three insecticides (**Table 4**).

**Table 3:** Effect of municipality and insecticide in the susceptibility of populations of *Helicoverpa* spp. at label rate recommendation.

Effect^1^	Df	F-value	p-value^2^
Insecticide	3	87.20	<0.0001^*^
Municipality	1	2.61	0.1211^n.s^
Insecticide vs Municipality	3	1.99	0.1463^n.s^

^1^ANOVA, Type III text of fixed effects.^2^ * statistically significant and n.s, not-statistically significant.

**Table 4:** Performance of *Helicoverpa* spp. populations at label rate concentrations of predominant insecticides adopted in high-input systems of field crop production in Puerto Rico.

Insecticide	Estimate mean mortality (%) ± CL (95%)^1^
Chlorantraniliprole	90.62 (81.36 – 99.99) a
Emamectin Benzoate	100.00 (100 – 100) a
Spinetoram	78.44 (11.31 – 41.32) b
Esfenvalerate	26. 32 (68.88 – 87.81) c

^1^ Larval mortality of Helicoverpa sp. corrected using Abbott’s formula ± Lower and upper **c**onfidence limits for the mortality means (95%).The same letters indicate the interval of confidence are not significantly different, p-value ≤ 0.05.

## Discussion

Trapping *Helicoverpa* spp. using sex pheromone lures over three years in high-input production systems of field crops, indicates the existence of year-round flight of this genus in Puerto Rico. A seasonal flight trend was also documented, with overall high moth abundance from October to March, and low abundance from April to September. However, even though the moths of this genus are strong fliers and able to spread throughout different regions (29), differences in moth abundance of *Helicoverpa* spp. between the municipalities in different months were significant. During the main crop season in Puerto Rico, which ranges from October to March, Juana Díaz had the highest number of moths caught through pheromone trapping. The municipality of Juana Diaz has one of the largest nursery breeding operations during what is defined as a winter season, from October to March, with high-input systems prevalently cultivated with corn. The agricultural landscape has also soybean, sunflower, and small acreages of sorghum (24). During the fallow season, which is usually from April to September, even though the landscape still has fields of soybean, sunflower, and tomato, the cultivated acreage drops drastically.

In polyphagous species, such as the genus *Helicoverpa*, the availability of different host plants has an important role in the increase of populations. In the United States, *H. zea* has a long history as an economic pest of cotton, but corn is the preferred agricultural host (30) together with sorghum, which also supports large populations of *H. zea* (31). In wild hosts, *H. zea* is associated with winter vetch, cranesbill, and crimson clover, and in Mississippi, these host plants are described as important early-season hosts. In North Carolina, toadflax and deergrass are reported as alternative hosts of *H. zea* (32, 33). *Helicoverpa armigera* also has a wide host plant range, including cultivated and wild plants, with over 172 and 200 host plants in Australia and China, respectively. In Japan, cotton (34, 35) and okra are recorded as top crops for *H. armigera* (35). In Brazil, in a study conducted post-invasion of *H. armigera*, the highest survival rates were recorded in soybean, cotton, and cowpea under laboratory conditions (36). The authors hypothesized that cultural practices create a shifting mosaic of habitats, in which populations of *H. armigera* can create a bridge using uncultivated crops during the fallow season (36), and survived in alternative cultivated hosts, such as green beans, tomatoes, citrus, and pastures (37). A high abundance of moths in Salinas was detected at the beginning of the fallow season, until June. In this municipality, during the crop season, the predominant crop is soybean, surrounded by other local farms, which entire operation are banana trees and other nursery small fields of soybeans and corn. During the fallow season, the region is predominantly cultivated with bananas, plantains, and scattered fields of sorghum. In addition, a remarkable presence of wild cotton is found around the perimeter of the cultivated fields, which during the fallow season is in the reproductive stage. The presence of this native cotton around the field during the autumn season may contribute to the maintenance of a higher abundance of *Helicoverpa* spp. detected during the pheromone trapping.

The results of male genitalia dissection and DNA analysis indicated only the presence of *H. zea* in the municipalities under study, even when the trapping used *H. armigera* pheromone lure. Closely related species of the genus *Helicoverpa* usually share common sex pheromone components with different ratios, and moths rely on these variations in multi-component pheromones to maintain reproductive isolation (38). A study conducted by Guerrero et al. (39) in two fields in Northern Florida from 2010 to 2011 showed that *H. zea* and *H. armigera* rubber septa lure with the same components (Suterra LLC, Bend, Oregon and Tréce, Inc., Adair, Oklahoma) resulted in 11,600 specimens collected and all specimens collected were identified as *H. zea* by male genitalia dissection. Cross-attraction has been documented in other species of lepidopteran pests, such as in the Plusiinae family, where the commercial formulation lure for *Chrysodeixis includens* also attracted *Ctenoplusia oxygramma, Rachiplusia ou, Trichoplusia ni*, and *Autographa verruca* (40).

In the present study, *H. armigera* was not detected in the samples of specimens that underwent genitalia dissection and molecular analysis. This result indicates the predominance of native *H. zea* in the landscape in the two municipalities under study. Previously, the documentation of the interaction and predominance of *H. zea* and the new invasive *H. armigera* in Brazil indicated that the proportion of each species was variable in different regions, and possibly due to differences in the agricultural landscapes (41). The aggressive behavior of *H. zea* when in intraspecific interaction with *H. armigera* and the variable acreages of corn and cotton were indicated as factors playing a role in the predominance of *H. zea* or *H. armigera* in different landscapes, respectively (41). In Puerto Rico, corn is the crop with large, cultivated acreage and may be favoring *H. zea*.

The genetic analysis for 550 specimens did not detect the presence of hybrids of *H. zea* and *H. armigera* in the pheromone trap collections from 2018 to 2021, providing no evidence for the expected field hybridization of these two species in Puerto Rico. Previously, the occurrence of hybridization between these two species was documented in the laboratory, under artificial conditions (1, 42). More recently, the possible interbreeding between *H. zea* and *H. armigera* has been indicated, based on the interspecific gene flow after *H. armigera* invasion in Brazil (43). The propensity of hybridization between these two species was later documented using whole-genome resequencing in combined samples from 16 countries, including specimens from Old World and South American populations (44). In addition, analysis with populations from high-input systems of field crops in Brazil estimated a range of 15 to 30% hybrid occurrence in the samples under analysis (45). The presence of natural hybridizations between *H. zea* and *H. armigera* in Puerto Rico is something expected due to the report of the invasive *H. armigera* in 2014. However, the population size of *H. armigera* in Puerto Rico is considered negligible (22) and possibly still not established (23), if compared to that in Brazil (46).

The relevance of analysis to detect hybridization between *H. zea* and *H. armigera* is due to the probability of new formations of adaptive genes (44, 47), which can have an impact on ecological attributes of the hybrids, such as a wider host range (48, 49) and performance of management tools (28, 50, 51). However, a study documenting biological parameters of hybrids between *H. armigera* and *H. zea*, under laboratory conditions, indicated that egg viability is a critical factor for the success of the hybridization, with overall egg viability of 14% (52), when compared with more than 85% egg viability in the parental species. The authors concluded that there are reproductive limitations for hybridization, including barriers from the lock-and-key mechanisms presented in the noctuid genitalia morphology (53, 54). It is expected that hybridization increases through both species’ abundance and time. The first report of *H. armigera* in Puerto Rico was in 2014, and the DNA analysis performed with samples from 2018 to 2021 did not detect hybrids in moths caught during the pheromone trapping. Future studies should keep monitoring for the expected presence of hybrids in Puerto Rico, especially focusing on specimens collected from corn fields, which is the predominant host crop in the agricultural landscape and represents an appropriate environment for intraguild interactions of both species (41).

The management of *Helicoverpa* spp. in Puerto Rico relies on larval scouting, moth trapping, crop rotation, the use of biopesticides, natural enemies, and insecticides. Due to the high pest pressure of lepidopteran pests, including *Helicoverpa* sp., there is a constant selection pressure for insecticide-resistant populations in Puerto Rico. During the crop season, the high-input systems of nursery production in Puerto Rico may require up to 30 insecticide applications in a 70 to 90-day crop cycle. The monitoring of the performance of insecticides with a different mode of action represents a critical aspect in an Insect Resistance Management (IRM) program, in an area-wide approach for the region, which has been implemented after failures to control lepidopteran pests in corn and soybean in Puerto Rico seed nurseries (55). The IRM program includes an approach of bi-monthly rotating insecticide modes of action, and then monthly rotations of insecticides to improve management (55). While this approach targeted pest species other than *Helicoverpa* sp., it effectively managed *Helicoverpa* populations at the time of application. The performance of four insecticides commonly adopted in this area wide IRM approach in Puerto Rico was tested considering 48 h of exposure in toxicological bioassays with larval mortality rate. The insecticides tested included chlorantraniliprole, which is a diamide (Coragen^®^, DuPont™) insecticide. This insecticide is a ryanodine receptor modulator causing contraction and paralysis in targeted pests (56). Emamectin benzoate (Proclaim^®^, Syngenta^®^) is an avermectin insecticide and acts as a glutamate-gated chloride channel allosteric modulator that causes paralysis (57). Spinetoram (Radiant^®^ SC, Corteva agriscience™) (27) is a spinosyn insecticide and is a nicotinic acetylcholine receptor allosteric modulator that causes hyperexcitation in the nervous system (Dow AgroSciences, 2006), and Esfenvalerate (Asana^®^ XL, Valent^®^) is a pyrethroid that acts as a sodium channel modulator (58)

The results of the performance of chlorantraniliprole, emamectin benzoate, spinetoram, and esfenvalerate indicated that there were no differences in the insecticide susceptibilities between populations collected in the two municipalities. The management approach adopted in both municipalities results in the same selection pressure in populations of *Helicoverpa* spp. However, significantly different performance of insecticides was detected, with the insecticides emamectin benzoate and chlorantraniliprole having a level of control above 90%. These insecticides should be considered in a rotation of mode of action program and efforts to keep tracking performance and susceptibility of populations of genus *Helicoverpa* in Puerto Rico. Spinetoram provided control below 70%, and esfenvalerate had lower efficacy in comparison to the other insecticides tested in *H. zea* with 30% or less larval mortality in populations of both municipalities. The use of spinosyns (Spinetoram and Spinosad) to manage *Helicoverpa* spp. has increased in recent years, with spinetoram being highly toxic to both species (23). In bioassays conducted by da Silva et al. (23), Spinetoram was highly toxic for both *Helicoverpa* species, *H. zea* and *H. armigera*, with spinetoram having an LC50 of 0.11-0.08 ug a.i./cm2. In the present study, spinetoram performance demonstrated a significantly lower performance when contrasted with emamectin benzoate and chlorantraniliprole. In addition, esfenvalerate had the lower performance among the four tested insecticides.

Resistance to pyrethroids in *H. zea* has been reported in Indiana and Illinois populations, along with others in the Midwest, northcentral, and northeastern populations (59), and more recently in the Florida Panhandle (60). Pyrethroid resistance in *H. armigera* has also been recorded in Benin and other West African countries (61). Increased cases of pyrethroid resistance in lepidopteran pest populations may lead to high adoption and consequently selection pressure of resistance to diamide insecticides (62). Diamides are relatively safe, and their biological, ecological, and toxicological attributes have high importance in an IRM program (62). The performance of the insecticides tested in this study, based on 48 h mortality with exposure to field rate diagnostic doses, indicated that three of the four insecticides commonly adopted in Puerto Rico should continue to be considered in a rotation to manage *Helicoverpa* spp. populations in field crop production in Puerto Rico.

In summary, the present study, performed in high-input nursery production of field crops in Puerto Rico, contributes to the IPM and IRM programs. The documentation of the phenology of flight of *Helicoverpa* spp. in high-input systems indicated that scouting activities in the fields should begin as early as October during the crop season. In addition, cultural control should be considered during the following season to eliminate volunteer plants or alternative hosts of *H. zea*, such as weeds, which may play a role in *Helicoverpa* spp. source of infestation during the crop season. The results of the bioassays indicated the differential performance of insecticides, which should be taken into consideration when selecting modes of action for rotation, in an IRM program. In addition, *H. zea* was the species detected in this study in commercial fields. However, there is a risk of the occurrence of hybrids with *H. armigera*, especially considering the possible increase of this invasive species, which can represent a challenge since management tools may perform differently. The occurrence of hybrids in the population along with the occurrence of *H. armigera* should be kept monitored, using updated molecular tools. Overall, the results of this work contribute with information to mitigation plans, in a scenario of an invasion of *H. armigera* in the continental U.S.

## Data availability statement

The raw data supporting the conclusions of this article will be made available by the authors, without undue reservation.

## Author contributions

All authors have contributed equally within specific sections of this manuscript. XLFR, SVPM, and JWJ contributed to the research design. XLFR conducted the experiments, and analyze the pheromone trapping, dissection, and bioassays data along with SVPM. OPP performed the molecular analysis. XLFR and SVPM wrote the manuscript, and JWJ and CJJ did the first English review, and all co-authors read and approved the manuscript.

## Funding

The publication fees will were supported by funds from NIFA USDA Hatch Project FLA-WFC-006203, Hatch Multistate project NC246 n. FLA-WFC-006003, and Multistate project S1080 FLA-WFC-006204. 

## Acknowledgments

Syngenta Seeds Puerto Rico for supporting the master’s program of the first author and the pheromone trapping data collection. Puerto Rico Agricultural Biotechnology Industry Association (PRABIA) for funds for molecular analysis. NIFA USDA Hatch Project FLA-WFC-006203, Hatch Multistate project NC246 n. FLA-WFC-006003, and Multistate project S1080 FLA-WFC-006204 partially funding supplies, and publications fees. Izailda B. dos Santos and Kelsey Hope for DNA extraction and analysis (UF/IFAS/West Florida Research Education Center, Jay, FL), and Calvin Pierce and Marissa Nufer (USDA Agricultural Research Service) Southern Insect Management Research Unit, Stoneville, MS) for technical assistance with molecular cloning and DNA sequencing.

## Conflict of interest

Author XLFR was employed by the company Syngenta Seeds. Author JWJ was employed by the company Syngenta Crop Protection Inc.

The remaining authors declare that the research was conducted in the absence of any commercial or financial relationships that could be construed as a potential conflict of interest.

## Publisher’s note

All claims expressed in this article are solely those of the authors and do not necessarily represent those of their affiliated organizations, or those of the publisher, the editors and the reviewers. Any product that may be evaluated in this article, or claim that may be made by its manufacturer, is not guaranteed or endorsed by the publisher.
